# Photochemical–Tissue Bonding Toward a More Effective Strategy for Corneal Epithelial Healing after Alkali Burn

**DOI:** 10.18502/jovr.v21.18256

**Published:** 2026-07-20

**Authors:** Salwa Abdelkawi, Shaimaa Mohammad, Dina Fouad, Mona Ebrahim, Aziza Ahmed

**Affiliations:** ^1^Biophysics and Laser Sciences Unit, Vision Science Department, Research Institute of Ophthalmology, Giza, Egypt; ^2^Department of Medical Application of Laser, Ophthalmic Unit, National Institute of Laser Enhanced Sciences, Cairo University, Giza, Egypt; ^3^Department of Medical Application of Laser, Photobiology Unit, National Institute of Laser Enhanced Sciences, Cairo University, Giza, Egypt

**Keywords:** Amniotic Membrane Grafting, Corneal Alkali Burn, Histological Evaluation, Image Analysis, Photochemical Tissue Bonding

## Abstract

**Purpose:**

This study evaluated the effectiveness of amniotic membrane photochemical–tissue bonding (AMPTB) versus amniotic membrane grafting (AMG) on treating corneal alkali burns in a rabbit model.

**Methods:**

Thirty-six albino rabbits with induced corneal alkali burns were divided into three groups: (a) untreated ulcer group (*n* = 12), (b) AMPTB group (*n* = 12), where the amniotic membrane (AM) was attached to the cornea using 532-nm light and Rose Bengal dye, and (c) AMG group (*n* = 12), in which the AM was secured using cyanoacrylate glue. The area, perimeter, and integrated density of the corneal ulcer were assessed using digital photographs analyzed with ImageJ software, alongside histological evaluation.

**Results:**

The AMPTB group experienced significant reductions in corneal ulcer area ( - 73.4%, - 79.8%, and - 89.3%), perimeter ( - 48.5%, - 54.0%, and - 67.2%), and integrated density ( - 81.2%, - 85.1%, and - 96.8%) after 1 day, 1 week, and 2 weeks, respectively. In comparison, the AMG group showed reductions in ulcer area ( - 65.2%, - 67.5%, and - 87.7%), perimeter ( - 35.5%, - 40.9%, and - 52.7%), and integrated density ( - 72.4%, - 69.5%, and - 94.7%). Additionally, histological analysis demonstrated earlier re-epithelialization of the cornea in the AMPTB group compared to the AMG group.

**Conclusion:**

The findings suggest that AMPTB promotes more rapid healing of the corneal epithelium and significant reductions in ulcer area, perimeter, and integrated density compared to AMG. AMPTB may, therefore, represent a promising alternative to AMG for the management of corneal alkali burns.

##  INTRODUCTION

Corneal ulcers are a major concern in ophthalmology.^[1,2]^ Traditional treatments for these ulcers include artificial tear drops, therapeutic contact lenses, amniotic membrane (AM) transplantation, and tarsorrhaphy. However, these approaches may not be sufficient for some patients, necessitating alternative strategies that promote epithelial proliferation and differentiation.^[3]^ Research highlights the effectiveness of the AM in experimental alkali burn models.^[4,5,6,7,8,9,10]^ The most common method for securing AM to the cornea is suturing, which can lead to complications such as infection, membrane dissolution, suture breakage, discomfort, bleeding, and tissue necrosis.^[11,12,13,14,15]^ Additionally, using cyanoacrylate glue to attach AM to corneal alkali burns has been shown to reduce edema and opacity, while improving wound healing by reducing inflammatory reactions and inhibiting collagenase production.^[9,16,17,18,19,20,21,22]^ Various studies have evaluated the effectiveness of 0.1% Rose Bengal (RB) and green-light activation, revealing a 72% success rate in preventing therapeutic corneal grafts in cases of resistant infectious keratitis.^[24,25,26]^

This study aims to compare corneal healing following alkali burns after treatment with AMPTB and AMG, examining the results in terms of ulcer area, perimeter, integrated density, and corneal histological structure at 1 day, 1 week, and 2 weeks post-treatment.

##  METHODS

### Experimental Animals

**Figure 1:**
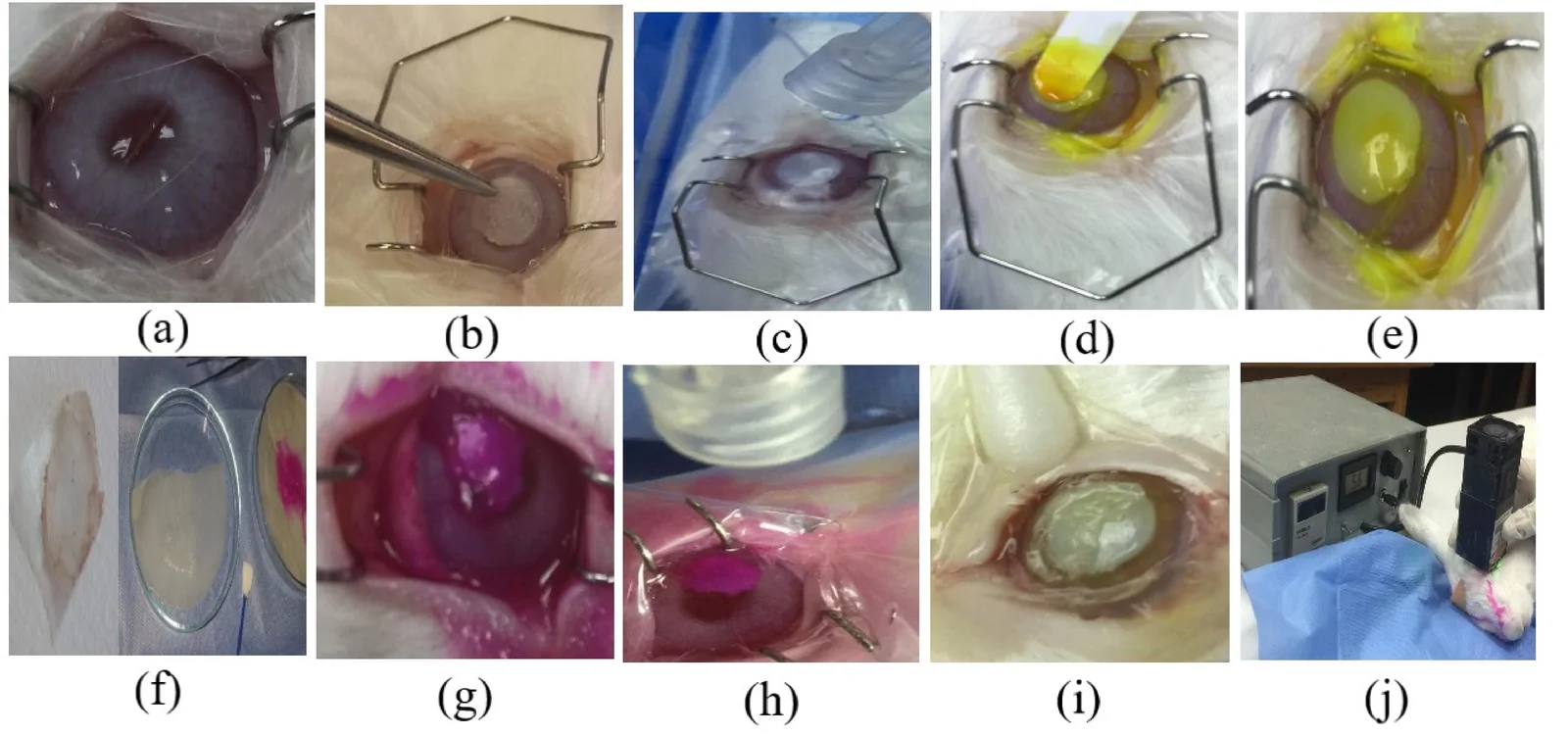
(a) Ocular exposure using a wire eyelid speculum. (b) A 5.0 mm diameter filter paper disc soaked in 1.0 N NaOH placed on the central cornea to induce a chemical burn. (c) Irrigation of the cornea with normal saline. (d & e) Fluorescein staining to demarcate the ulcerated area. (f) Application of 0.1% RB solution to the stromal surface of the AM for 5 minutes. (g & h) Staining of the corneal ulcer bed with 1% RB followed by saline irrigation. (i) Smoothing and apposition of the AM over the ulcer. (j) Photochemical tissue bonding of the AM to the corneal surface using a 532 nm diode-pumped solid-state laser.

Thirty-six female New Zealand rabbits (2.0-2.5 kg) were maintained under standard laboratory conditions with a 12-hour light/dark cycle and unrestricted access to food and water. The experimental protocol was approved by the Institutional Animal Care and Use Committee of Cairo University, Egypt (CU-IACUC; approval no.1/F/30/19). All procedures were conducted in accordance with the guidelines of the Association for Research in Vision and Ophthalmology (ARVO) and the National Institutes of Health (NIH) for the use of laboratory animals in vision and ophthalmic research. Prior to the experiment, eye examinations—including slit-lamp biomicroscopy, indirect ophthalmoscopy, and fluorescein staining—were performed on both eyes to confirm the absence of infection, edema, or inflammation. The rabbits (*n* = 36) were anesthetized intramuscularly with 0.2 mL/kg xylazine (Xyla-Ject) and 0.6 mL/kg ketamine hydrochloride (Sigma Chemical Co., St. Louis, Missouri, USA). For local anesthesia, 0.5% topical proparacaine hydrochloride (Alcon Laboratories, Inc., Fort Worth, Texas, USA) was applied, and a lid speculum was used to expose the corneal surface [Figure 1a].

Corneal ulcers were induced by applying a 5.0-mm diameter Whatman #3 circular filter-paper disc soaked in 1.0 N NaOH solution to the right cornea for 30 seconds [Figure 1b]. The eye was immediately rinsed with 0.9% normal saline solution for 2 minutes [Figure 1c]. The ulcerated area was then delineated using fluorescein dye [Figures 1d & 1e]. The rabbits were randomly divided into three groups: ulcer group (*n* = 12), amniotic membrane photochemical-tissue bonding (AMPTB) group (*n* = 12), and amniotic membrane grafting (AMG) group (*n* = 12).

Photographs of the corneas were taken with a slit-lamp camera adaptor (Digital Eye Center, Miami, Florida, USA) after 1 day, 1 week, and 2 weeks post-burn. In the AMG group, most of the AM was detached by day 6, whereas in the AMPTB group, most of the detachment occurred around day 9.

### Amniotic Membrane Photochemical-tissue Bonding (AMPTB) 

The experiment was performed on the same day as ulcer induction. The timeline for the experiment, from ulcer induction to treatment, followed previously published protocols.^[13,14]^ A sterile human AM patch (Bio Tissue Corporation, Miami, USA) was placed on filter paper with the stromal side facing upward and treated with 0.1% RB dye in phosphate-buffered saline (P Sigma-Aldrich, St. Louis, Missouri, USA) [Figure 1f]. The ulcerated cornea was treated with 1% RB dye in PBS [Figure 1g]. After 2 minutes, the cornea was rinsed with a 0.9% saline solution [Figure 1h]. Following a 5-minute interval, the dye-stained AM patch was trimmed to appropriate dimensions to cover the ulcerated cornea, with the epithelial basement membrane side facing up. All wrinkles were gently smoothed with a cotton swab [Figure 1i].

A diode-pumped solid-state laser with a continuous wavelength (CW) of 532 nm (DPSS, HHs6-2, Tim e-relay, Elexperc, Egypt), designed for laboratory applications, was used to bond the stromal surface of the AM to the cornea. The laser parameters were set at an irradiance of 0.25 W/cm² and a fluence of 100 J/cm², delivered over 6.7 minutes, according to the equation:

Fluence (J/cm²) = Irradiance (W/cm²) × Time (s).

The laser beam was transmitted through a 4-mm-diameter optical fiber [Figure 1j]. During irradiation, the AM surface was gently moistened with distilled water every 90 seconds.

### Amniotic Membrane Grafting (AMG) 

**Figure 2:**
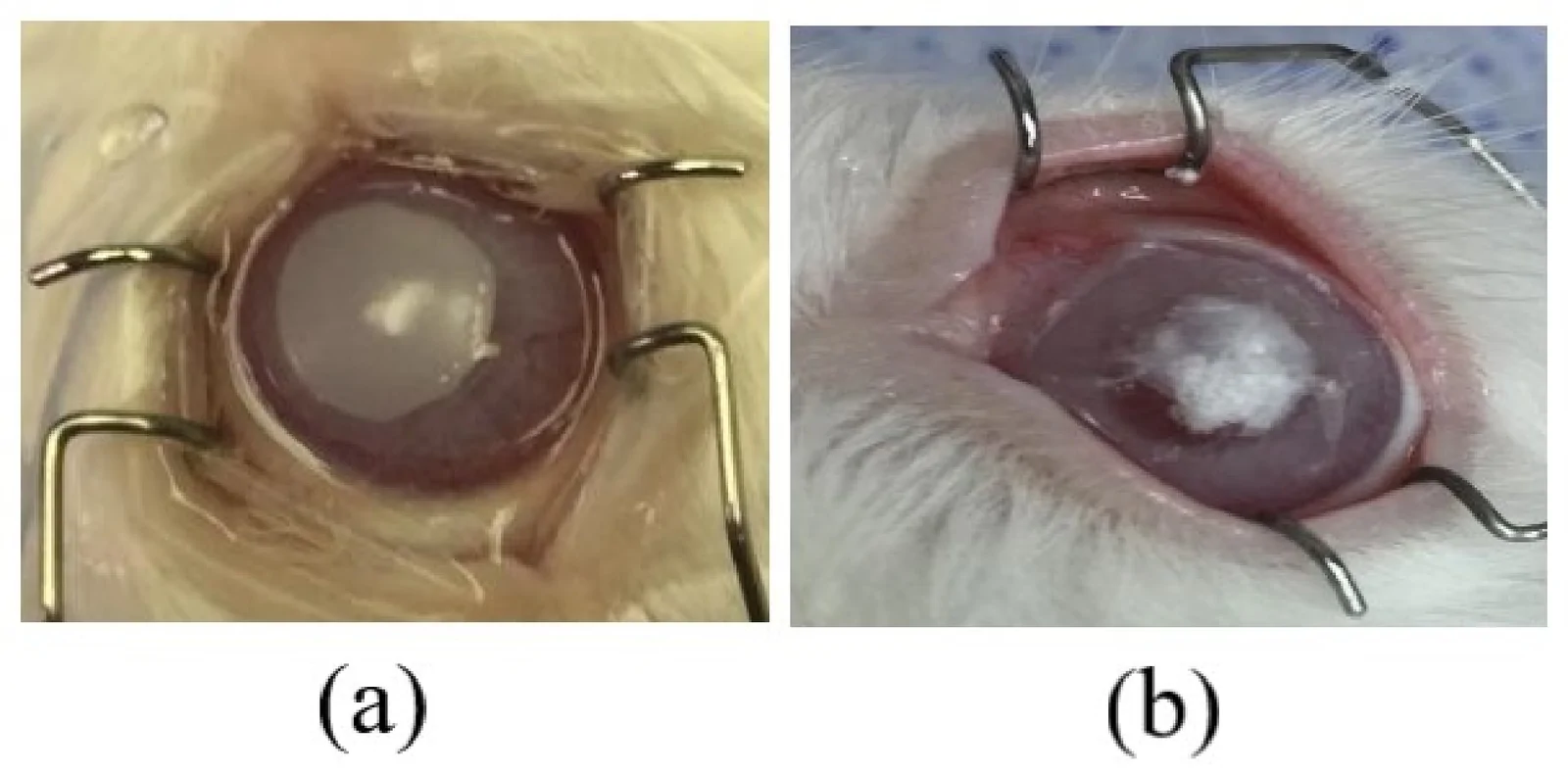
(a) Ocular exposure and debridement of loose epithelial tissue from the ulcerated cornea. (b) Application of cyanoacrylate glue followed by placement of the AM (stromal side down) onto the treated area.

The ulcerated cornea was cleaned and debrided with a sterile cotton swab to remove the loose or dead epithelial tissue. A lid speculum was carefully inserted to expose the corneal surface [Figure 2a]. A very small quantity of cyanoacrylate glue (monomeric n-butyl-2-cyanoacrylate, Histoacryl, B. Braun Surgical SA, Rubi, Spain) was applied using a 1-mL syringe without a needle to slightly cover the ulcerated area and extend beyond its margins, ensuring fixation of the AM edges while avoiding contact with the nictitating membrane or eyelids. An appropriately sized AM was then positioned over the glued area [Figure 2b].

Following the procedure, rabbits were housed for 24 hours in cages with circular openings to prevent eye rubbing and allow sufficient time for membrane adhesion to the cornea. Postoperatively, topical antibiotics and steroid eye drops (a combination of tobramycin and dexamethasone, Tobradex, Alcon, USA) were administered.

### Histological Examination of the Cornea

Four rabbits from each group were euthanized with IV injections of ketamine hydrochloride and xylazine hydrochloride (5X the anesthetic dose) 1 day, 1 week, and 2 weeks after treatment. The eyes were instantly enucleated and initially fixed in 2.5% glutaraldehyde in phosphate buffer for 30 minutes. The corneas were then carefully excised from the anterior segment for histopathological examination.

Corneal samples were dissected into small pieces and instantly fixed in phosphate-buffered glutaraldehyde (2.5%, pH 7.4) at 4ºC for 6 hours. The specimens were subsequently post-fixed in 1% osmium tetroxide for 60 minutes, dehydrated in ethanol, and embedded in an Araldite CY212 resin mixture. Semithin sections (1.0 μ m) were prepared and stained with toluidine blue for microscopic examination. The samples were processed and analyzed by a specialist in the Histology Unit at the Research Institute of Ophthalmology, Giza, Egypt.

### Statistical Analysis

The results for the area, perimeter, and integrated density were expressed as the mean ± standard deviation (SD). Statistical analysis was performed using Student's *t-*test for unpaired data, while comparisons among multiple groups were conducted using analysis of variance (ANOVA). Statistical analyses were performed using SPSS version 11 for Windows (IBM Inc., Chicago, Illinois, USA), with a significance level set at *P* < 0.05.

##  RESULTS

**Table 1:** The area, perimeter, and integrated density of the ulcerated cornea after treatment with AMPTB and AMG, measured by ImageJ software

**Group**	**Area (mm^2^)**	**Change (%)**	**Perimeter (mm)**	**Change (%)**	**Integrated density**	**Change (%)**
Ulcer	339 ± 19.6	–	65.3 ± 7.0	–	37.7 × 10^2^	
AMPTB 1 day	90.2 ± 14.6 ${}^{*}$	–73.4	33.6 ± 5.4 ${}^{*}$	–48.5	7.1 × 10^2^	–81.2
AMPTB 1 week	68.6 ± 8.25 ${}^{*}$	–79.8	30.0 ± 3.9 ${}^{*}$	–54.0	5.6 × 10^2^	–85.1
AMPTB 2 weeks	36.4 ± 7.39 ${}^{*}$	–89.3	21.4 ± 3.0 ${}^{*}$	–67.2	1.2 × 10^2^	–96.8
AMG 1 day	118 ± 22.0 ${}^{*}$	–65.2	42.1 ± 6.0 ${}^{*}$	–35.5	10.4 × 10^2^	–72.4
AMG 1 week	110 ± 29.8 ${}^{*}$	–67.5	38.6 ± 3.5 ${}^{*}$	–40.9	11.5 × 10^2^	–69.5
AMG 2 weeks	41.5 ± 8.43 ${}^{*}$	–87.7	30.9 ± 4.9 ${}^{*}$	–52.7	1.9 × 10^2^	–94.7
${}^{*}$ Significant difference when compared with the ulcer group; the values are expressed as mean ± SD; SD, standard deviation.						

**Figure 3:**
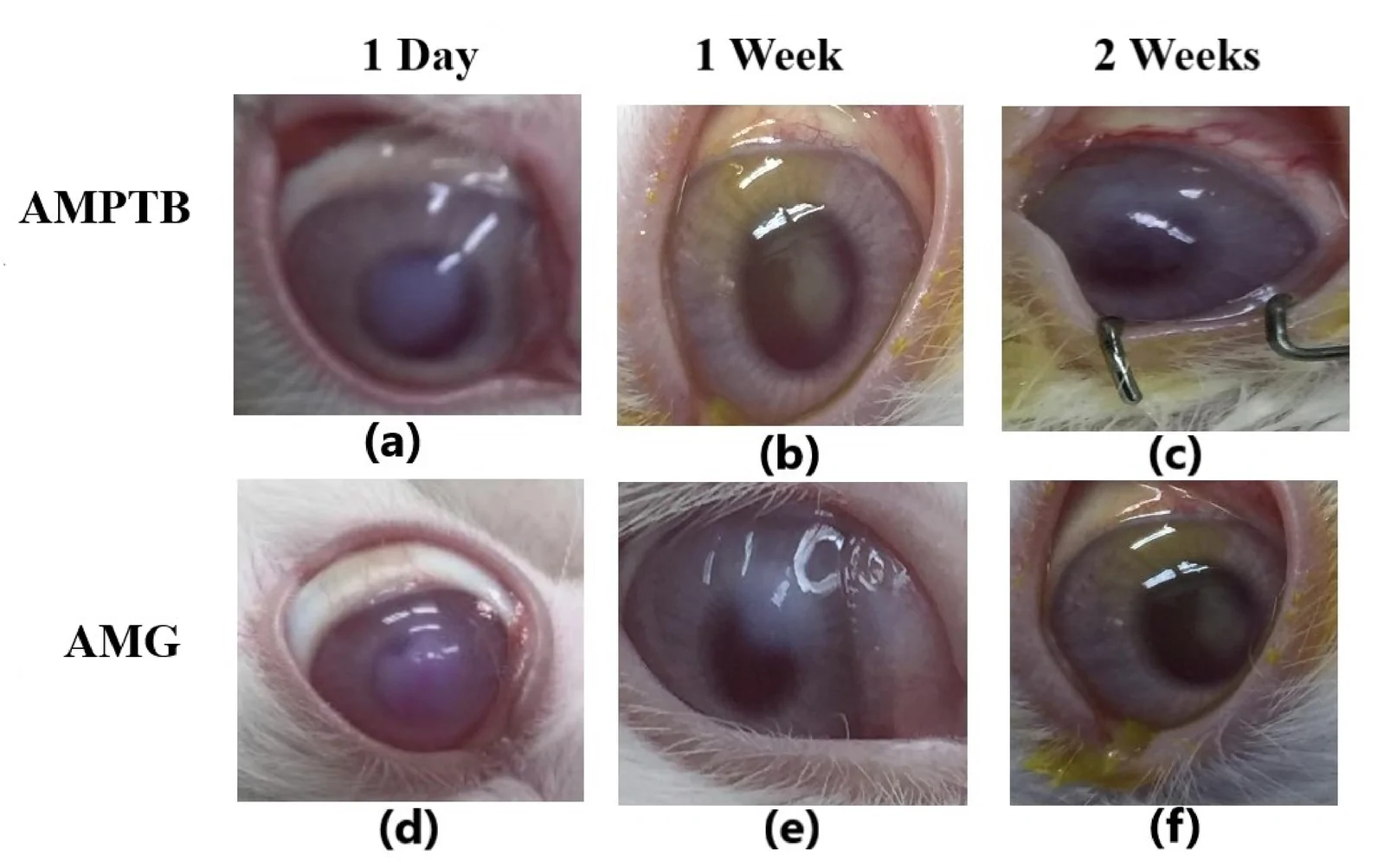
Representative photographs showing the progression of corneal healing following a 1.0 N NaOH alkali burn. Note the minimal opacities in the AMPTB group (a–c) compared with the AMG group (d–f) 1 day, 1 week, and 2 weeks post-treatment.

**Figure 4:**
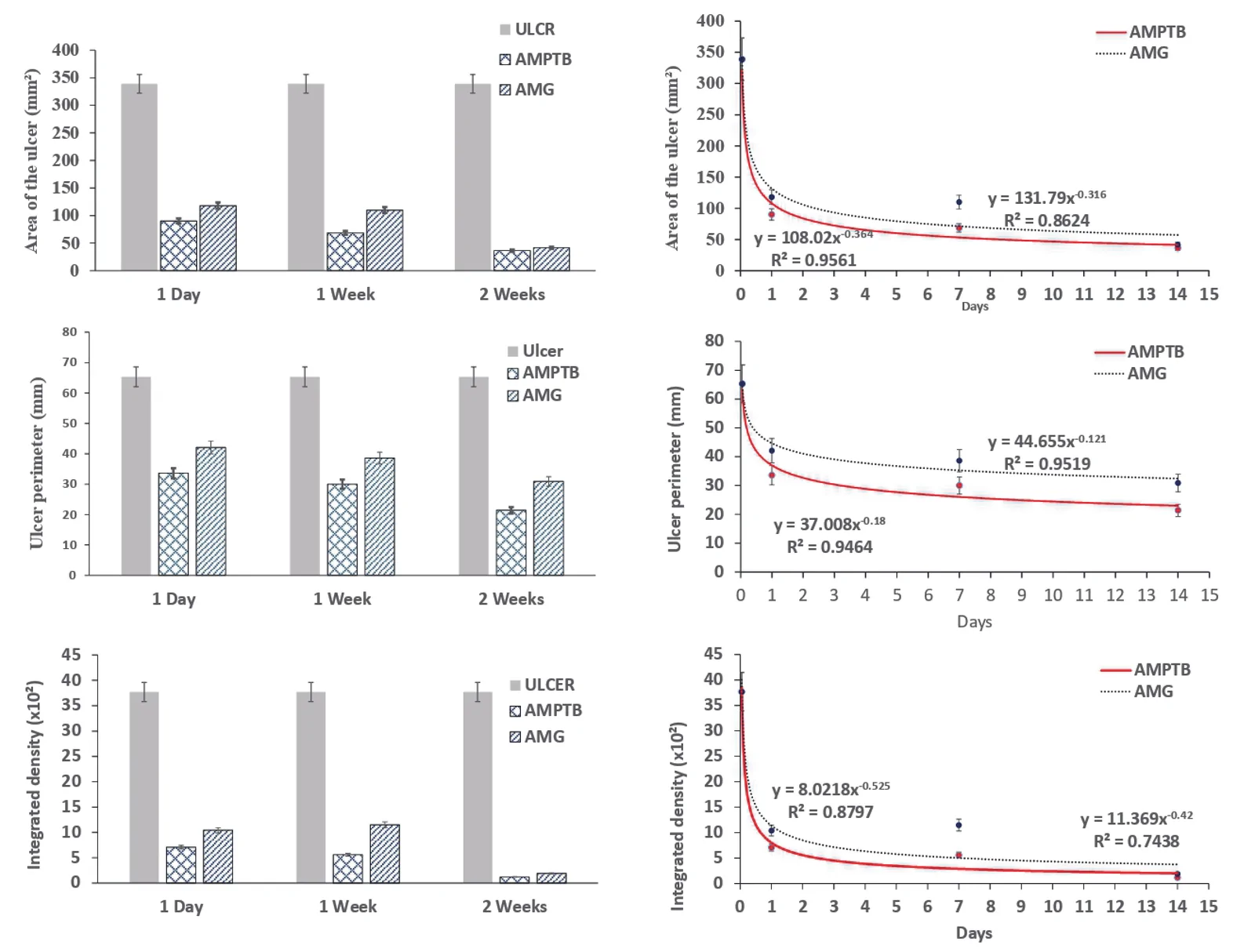
(a, c, e) Longitudinal changes in ulcer area, perimeter, and integrated density. (b, d, f) Power-fitting regression curves representing the rates of change for each parameter over time for the AMPTB and AMG groups.

### ImageJ Analysis Results

Central alkali burns were induced in rabbit corneas by applying circular filter paper discs soaked in 1.0 N NaOH [Figures 1b & 2a]. The gradual healing of corneal ulcers following treatment with AMPTB and AMG was documented photographically at three time intervals [Figure 3]. After 2 weeks, the AMPTB group demonstrated clearer corneas with minimal opacity compared with the AMG group. Moreover, ulcers treated with AMPTB showed better restoration of corneal integrity than those treated with AMG. No corneal perforation or scarring was observed, and only insignificant superficial punctate keratitis with a relatively smooth corneal surface was noted. Follow-up revealed no statistically significant differences between the two treated groups; however, mild and nonsignificant corneal neovascularization was observed in the AMG group after 1 day.

Quantitative analysis using ImageJ software was performed to determine ulcer area, perimeter, and integrated density over time [Table 1; Figure 4]. In the AMPTB group, the mean ulcer area following alkali burn was 339 ± 19.6 mm², showing significant reductions of - 73.7%, - 79.8%, and - 89.3% after 1 day, 1 week, and 2 weeks, respectively [Table 1]. The AMG group also demonstrated progressive improvement over time [Figure 4a], with reductions in ulcer area of - 65.2%, - 67.5%, and - 87.7% at the same time points [Table 1].

The mean perimeter of the ulcerated area before treatment in the AMPTB group measured 65.3 ± 7.0 mm, exhibiting a decrease of - 48.5%, - 54.4%, and - 67.2% after 1 day, 1 week, and 2 weeks, respectively [Figure 4c]. In the AMG group, the perimeter decreased by - 35.5%, - 40.9 %, and - 52.7% after 1 day, 1 week, and 2 weeks, respectively [Table 1]. Integrated density, which measures the pixel intensity in the region of interest (ROI) compared with the surrounding intact cornea, also improved over time. In the AMPTB group, integrated density decreased by - 81.2%, - 85.1%, and - 96.8% after 1 day, 1 week, and 2 weeks, respectively [Figure 4e; Table 1]. Similarly, the integrated density for the AMG group indicated a noticeable decrease of - 72.4%, - 69.5%, and - 94.7% at the corresponding time points [Figure 4e; Table 1].

Regression analysis demonstrated a strong correlation between ulcer healing and time in the AMPTB group, with R² values of 0.96, 0.95, and 0.88 for ulcer area, perimeter, and integrated density, respectively [Figures 4b, 4d, & 4f]. In the AMG group, the corresponding R² values were 0.86, 0.95, and 0.74 [Figures 4b, 4d, & 4f].

### Histological Evaluation of the Corneal Healing

**Figure 5:**
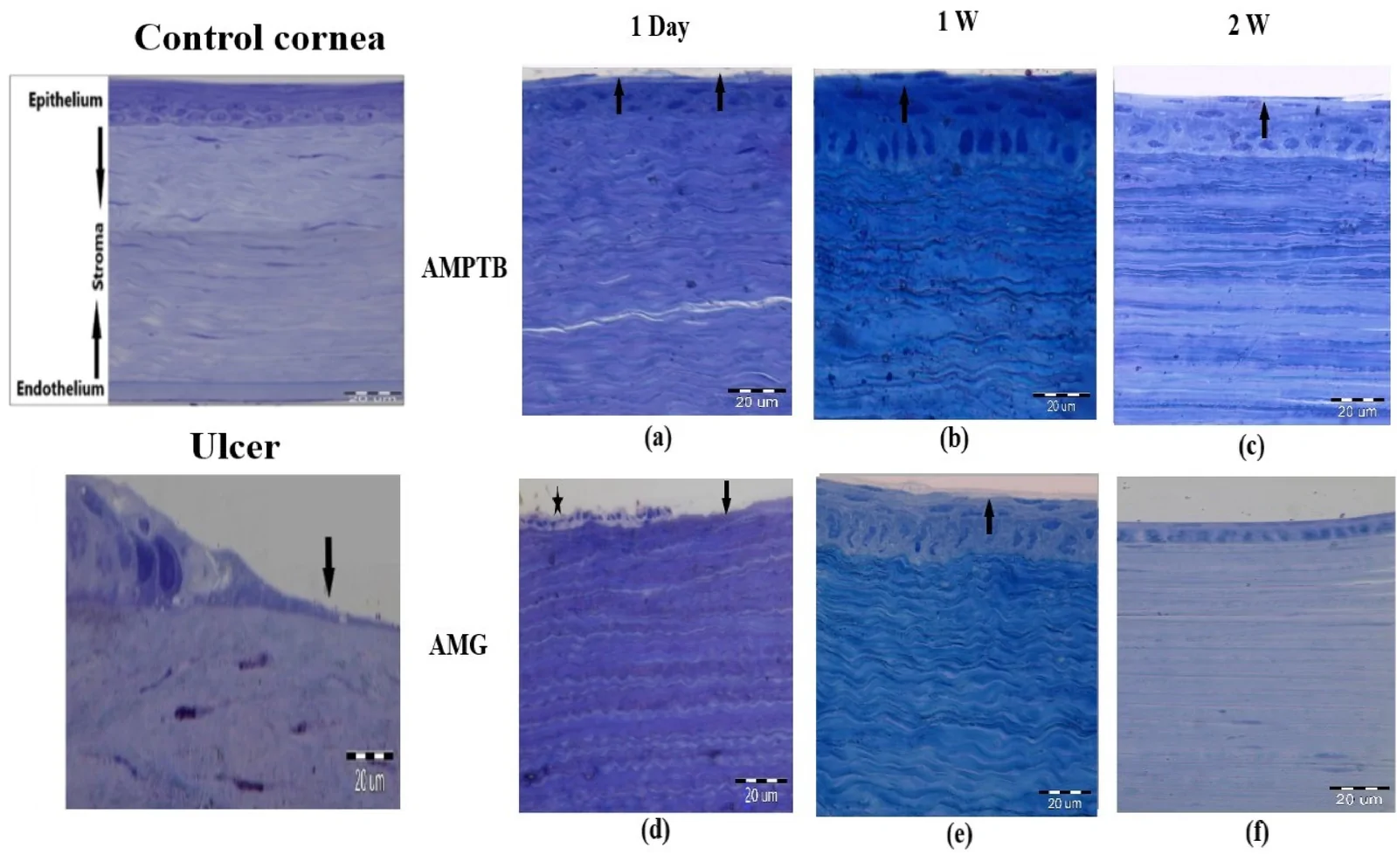
Light micrographs of corneal sections (H&E staining). The control cornea (upper left) exhibits intact layers, while the untreated ulcerated cornea (lower left) shows epithelial loss and absence of basal cells (arrow). AMPTB group: (a) at day 1 showing presence of basal cells with loss of superficial epithelial layers (arrows); (b) at 1 week showing significant epithelial regeneration; (c) at 2 weeks showing complete epithelial recovery. AMG group: (d) at day 1 showing localized basal cells (asterisk) with persistent ulceration elsewhere (arrow); (e) at 1 week showing improved basal cell organization with a slight persistent epithelial defect; (f) at 2 weeks showing wound closure [T. BX500].

A light microscopic photograph of the control cornea revealed intact corneal epithelium and a normal, well-organized layered architecture [Figure 5]. The cornea exposed to 1.0 N NaOH exhibited an ulcerated condition, characterized by the loss of epithelium and basal cells (arrow) [Figure 5]. Epithelial healing was assessed after treating the ulcers with AMPTB and AMG over three successive periods. After 1 day of treatment with AMPTB, the basal cells had appeared, although the normal epithelial architecture was not yet restored (arrows) [Figure 5a]. Moreover, this group showed an improved epithelial layer after 1 week [Figure 5b], followed by complete epithelial recovery after 2 weeks [Figure 5c].

On the other hand, after 1 day, the histological examination of corneas treated with AMG revealed a localized regeneration of basal cells (star), while an ulcerative area persisted on the opposite side (arrow) [Figure 5d]. Furthermore, the histological analysis indicated improvement in the basal cells after 1 week, with a small ulcerative area still visible within the epithelial layer [Figure 5e], followed by complete healing after 2 weeks [Figure 5f].

##  DISCUSSION 

Alkali burns represent one of the most severe chemical injuries affecting the anterior segment of the eye.^[4]^ Repairing a severely ulcerated cornea poses a significant challenge for ophthalmologists. In recent years, various methods and materials have been documented to address corneal defects.^[4,5,8,10,19,27,28]^ Despite the increasing emphasis on medical treatments, moderate to severe alkali burns remain difficult to manage, often resulting in extended treatment durations and multiple complications. The effective management of ocular chemical burns focuses on enhancing epithelialization, minimizing inflammation, and preventing complications. Primary treatment options include artificial tears, topical steroids, ascorbate, and biological medications. Early amniotic membrane treatment (AMT) is beneficial for reducing inflammation and promoting tissue healing, while limbal stem cell transplantation (LSCT) is effective in cases of extensive conjunctivalization.^[29]^ For grade I and II burns, treatment typically includes frequent lubrication, topical corticosteroids, antibiotics, systemic doxycycline, and vitamin C, with preservative-free medications recommended. In the case of grade III burns, 20% autologous serum should be incorporated into the standard treatment, necessitating daily follow-up. For grades IV to VI, AMT, with or without tenoplasty, is recommended, highlighting the need for inpatient care.^[29]^

Photochemical tissue bonding (AMPTB) technology effectively attaches the AM to the surface of the cornea by forming molecular crosslinks between proteins. The resulting seal is both immediate and robust enough to close penetrating eye injuries, with seal power increasing as fluence increases, while requiring only a short irradiation time. The sutureless technique offers benefits such as speed, immediate watertight seal, and a reduced risk of corneal injury compared with sutures.^[30]^ Applying cyanoacrylate glue to the cornea to secure the AM is a simple procedure that minimizes bleeding and may help reduce inflammation. This glued AMT provides a painless alternative to traditional sutured techniques, potentially eliminating the need for general anesthesia and allowing more rapid treatment.^[12]^ While fibrin glue has been used to seal the AM to the ocular surface and is appropriate for low-tension situations, clinical experience indicates that it may not provide the necessary bonding strength for more significant lacerations.^[30]^

This study was conducted to address the lack of prior research comparing the effects of AMG with cyanoacrylate and AMPTB in experimental animal models. Attaching the AM via suturing demands considerable surgical expertise and has notable disadvantages. These include prolonged operating times and complications such as abscesses, granuloma, and tissue necrosis. Patients may experience corneal irritation, scarring, graft loss due to membrane shrinkage, and pain. A potential alternative to sutures could be bio-adhesives, which might mitigate some of these issues.^[13]^

Using sutureless AM with cyanoacrylate as a dressing for corneal alkali burns may promote rapid epithelial healing with reduced vascularization and greater persistence compared to AM suturing, representing a significantly faster and more beneficial approach.^[13]^ The drawbacks of this approach include an elevated risk of infection, patient discomfort, and the possibility of glue dislodging if the surface is uneven.^[14]^

In this investigation, following alkali burns, corneal opacity was significant and correlated with edema resulting from fluid retention and the disruption of the regular collagen lamellae pattern in the ulcer group.^[31]^ After the alkali burn, limbal capillaries facilitated the entry of polymorphonuclear leukocytes (PMNs) into the wound as they migrated through the stroma, leading to recurring epithelial defects.^[32]^ Multiple proteinases resulting from the damaged cornea may contribute to ulcer progression.^[33]^

Examining epithelial healing in response to alkaline burns helps identify innovative therapeutic options. The corneal epithelium primarily heals through the migration of adjacent cells, accompanied by cell flattening and an increase in size and volume.^[34,35,36]^ Cells at the wound margin are limited in movement, and the reorganization of the epithelial layer occurs behind the wound edge. The underlying substrate may also help trigger the healing process.^[37,38]^

Treatment of persistent epithelial defects with AM has been shown to promote epithelialization, potentially through the inhibition of collagenase and the provision of growth factors and a basement membrane.^[39]^ In cases of corneal alkali burns, AM prevents polymorphonuclear neutrophil (PMN) infiltration, thereby reducing local inflammatory responses and improving wound healing.^[40]^ Furthermore, the basement membrane of AM promotes epithelial differentiation and growth, enhances the adhesion of basal epithelial cells, and reduces apoptosis.^[9]^

The AM stromal matrix significantly reduces the levels of the potent proinflammatory cytokines IL-1 α and IL-1 β , thereby decreasing inflammation.^[40,41]^ Moreover, components present in AM, including secretory leukocyte proteinase inhibitors (SLPI), β -defensin (antimicrobial peptides), and elafin, exhibit antimicrobial properties.^[40,42,43]^

This study evaluated the healing effectiveness of corneal ulcers in rabbits subjected to alkali burns, focusing on treatment outcomes with AMPTB and AMG across three time intervals. The colored photographs clearly demonstrated that corneas in the AMPTB and AMG treatment groups improved significantly with minimal opacity, providing encouraging evidence of their effectiveness compared with the other ulcerated group.

We employed ImageJ analysis software to assess the healing of the ulcerated cornea by measuring wound area, perimeter, and integrated density. These quantitative parameters reflected the remaining wound area during the healing period in all treated groups. Statistically significant differences (*P* < 0.001) were noted following treatment with AMPTB and AMG. Additionally, the data presented in Table 1 indicate that the AMPTB group showed earlier improvement than the AMG group; the area percentage change after 1 day was - 73.4% for AMPTB versus - 65.2% for AMG. Furthermore, perimeter and integrated density exhibited similar trends after 1 day, 1 week, and 2 weeks. In this study, we utilized power-fitting curves to assess the performance of both treatment models. Power-curve plotting allowed direct comparison between the two treatment methods and facilitated evaluation of their therapeutic effectiveness. Analysis of the curve shape and regression parameters showed that a sharp regression (R^2^) reflects a strong likelihood of improvement over time. Furthermore, the curve fitting results presented in Figures 4b, 4d, and 4f, together with their R² values, provide compelling evidence of earlier improvement in the AMPTB group compared with the AMG group.

The histological examination of the cornea confirmed the findings from the ImageJ analysis, demonstrating epithelial regeneration, wound closure, and restoration of an intact corneal epithelium [Figure 5]. These results support previous studies suggesting that a combination of AM and cyanoacrylate (AMG) could enhance corneal healing and structural organization.^[7]^ Thus, human AM possesses anti-angiogenic and anti-inflammatory properties (including endostatin, thrombostatin-1, and interleukin-1 receptor antagonists), while cyanoacrylate provides antimicrobial effects and inhibits collagenase production.^[7,17,22,18,44]^ In ophthalmic surgery, cyanoacrylate derivatives are the most commonly used glues for repairing corneal perforations and treating corneal diseases.^[14,18,45]^

Cyanoacrylate tissue adhesive was intentionally applied in a very small quantity over the corneal ulcer before placement of the AM. This reflects the standard and widely accepted sutureless clinical approach for managing small corneal perforations and non-healing ulcers. When applied as a thin, controlled layer, cyanoacrylate polymerizes to form a firm, protective barrier that seals the defect, prevents aqueous leakage, and serves as a temporary scaffold that facilitates epithelial migration from the surrounding healthy cornea rather than inhibiting it.^[14,45,46,47]^ Although cyanoacrylate is in direct contact with the corneal surface, multiple studies have confirmed that, when properly applied, it supports epithelial healing, maintains corneal contour, and reduces the risk of secondary infection.^[14,45,46,47]^

Cyanoacrylates adhere to the surface within 5-6 seconds in the presence of water and finally undergo exothermic polymerization within 60 seconds.^[45,48]^ The mechanism of action resembles the granulation process occurring during various stages of wound healing.^[49]^ N-butyl-2-cyanoacrylate has been widely used as a medical sealant for wound healing, and its butyl ester form provides firm and rigid bonding.^[50]^ N-butyl-2-cyanoacrylate is characterized by long-chain molecular structures, and when it comes into contact with blood or water in tissue, it forms a very tight protective layer.^[51]^ It effectively binds to tissue and exhibits bacteriostatic effects, and a hydrolytic process gradually removes the layer formed by the glue.^[50]^ However, long-chain forms degrade slowly with minimal toxic effects and can be easily used in topical applications.^[48]^ Furthermore, it provides tectonic support, has a high tensile strength, creates a strong bridge between wound margins, and arrests the progression of keratolysis.^[51]^

Consistent with earlier research findings, corneal healing and epithelial wound closure were achieved in the AMPTB group without complications.^[26]^ Moreover, healing in the AMPTB group occurred more rapidly than in the AMG group after 1 week [Figures 5b & 5e]. This acceleration may be attributed to the superficial penetration of RB dye, which strongly associates with stromal collagen and exhibits short-range effects due to photoactivation.^[52,53,54]^ Additionally, prior studies have shown a strong bond between RB and collagen, facilitated by the negative enthalpy of RB-collagen binding (collagens type I and III contain positively charged lysine and arginine residues that can form ionic bonds with the negatively charged RB).^[23,54]^ The treated area, exposed to a green laser, activates the RB dye, initiating the formation of molecular covalent crosslinks that connect proteins across tissue surfaces through a photochemical, nonthermal mechanism.^[55]^ This process is independent of cell mitosis but involves functional actin filaments and focal adhesion sites.^[56,57,58]^

Future studies may consider additional quantitative endpoints, such as histomorphometric scoring of epithelial thickness, stromal organization, and inflammatory cell infiltration; scoring of corneal transparency or opacity; and immunohistochemistry for epithelial or inflammatory markers.

In summary, this study demonstrated that both AMPTB and AMG resulted in improvements in corneal ulceration; however, the AMPTB group showed greater reduction in area, perimeter, and integrated density compared with the AMG group. These results indicate that AMPTB offers a safe, effective, and noninvasive method for treating corneal ulcers, eliminating the need for surgical intervention, sutures, or glue-associated complications. The findings also support the safety of RB and green laser (532 nm) photochemical tissue bonding. Therefore, it may be considered as a potential alternative for treating alkali burn-induced corneal ulcers.

##  Financial Support and Sponsorship

None.

##  Conflicts of Interest

None.
